# BK channels promote action potential repolarization in skeletal muscle but contribute little to myotonia

**DOI:** 10.1007/s00424-024-03005-z

**Published:** 2024-08-16

**Authors:** Chris Dupont, Brianna Blake, Andrew A. Voss, Mark M. Rich

**Affiliations:** 1 Department of Neuroscience, Cell Biology and Physiology, Wright State University, Dayton, OH 45435 USA; 2 Department of Biological Sciences, Wright State University, Dayton, OH 45435 USA

**Keywords:** Excitation, Myotonia congenita, t-tubule, K^+^ channel, Potassium

## Abstract

Patients with myotonia congenita suffer from slowed relaxation of muscle (myotonia), due to hyperexcitability caused by loss-of-function mutations in the ClC-1 chloride channel. A recent study suggested that block of large-conductance voltage- and Ca^2+^- activated K^+^ channels (BK) may be effective as therapy. The mechanism underlying efficacy was suggested to be lessening of the depolarizing effect of build-up of K^+^ in t-tubules of muscle during repetitive firing. BK channels are widely expressed in the nervous system and have been shown to play a central role in regulation of excitability, but their contribution to muscle excitability has not been determined. We performed intracellular recordings as well as force measurements in both wild type and BK^−/−^ mouse extensor digitorum longus muscles. Action potential width was increased in BK^−/−^ muscle due to slowing of repolarization, consistent with the possibility K^+^ build-up in t-tubules is lessened by block of BK channels in myotonic muscle. However, there was no difference in the severity of myotonia triggered by block of muscle Cl^−^ channels with 9-anthracenecarboxylic acid (9AC) in wild type and BK^−/−^ muscle fibers. Further study revealed no difference in the interspike membrane potential during repetitive firing suggesting there was no reduction in K^+^ build-up in t-tubules of BK^−/−^ muscle. Force recordings following block of muscle Cl^−^ channels demonstrated little reduction in myotonia in BK^−/−^ muscle. In contrast, the current standard of care, mexiletine, significantly reduced myotonia. Our data suggest BK channels regulate muscle excitability, but are not an attractive target for therapy of myotonia.

**Supplementary Information:**

The online version contains supplementary material available at 10.1007/s00424-024-03005-z.

## Introduction

Myotonia congenita is an inherited skeletal muscle disease caused by loss-of-function mutations of ClC-1 chloride channels [20, 21, 35]. Cl^−^ conductance in muscle serves to stabilize the resting membrane potential near the Cl^−^ equilibrium potential [1, 35]. Loss of Cl^−^ channel function leads to muscle hyperexcitability, which results in myotonia (inability to relax muscle) due to myotonic discharges (involuntary firing of action potentials) [17].

A recent study suggested that block of large-conductance voltage- and Ca^2+^- activated K^+^ channels (BK, Maxi-K, slo1, or KCa1.1 channels) may be an attractive target for development of therapy for myotonia congenita [19]. BK channels are widely expressed in the nervous system where they play a central role in repolarization of the action potential [6, 33, 45]. BK channels are expressed in skeletal muscle [14, 39] and mice lacking functional BK channels have motor dysfunction [9, 18, 24, 34, 40]. The connection between muscle BK expression and muscle function is not clear. For example, we found that an important contributor to weakness in BK^−/−^ muscle was decreased vesicle release at the neuromuscular junction rather than muscle dysfunction [42].

An important contributor to triggering of myotonic discharges is K^+^ build-up in t-tubules. T-tubules are small invaginations in skeletal muscle that serve to conduct action potentials (APs) to the center of fibers. K^+^ build-up in t-tubules occurs as K^+^ exits muscle fibers during the repolarization phase of APs [1, 2, 8, 16, 41]. The build-up shifts the Nernst potential for K^+^ to more depolarized values, which depolarizes the membrane potential and is thought to drive myotonic discharges. In normal muscle, ClC-1-mediated Cl^−^ current offsets the depolarizing influence of K^+^ build-up in t-tubules and helps drive K^+^ back into muscle fibers [2, 8, 41]. A recent study by Hoppe et. al. proposed that BK channels promote myotonic discharges by contributing to K^+^ build-up in t-tubules [19].

Motivated by the suggestion that BK channels contribute to generation of myotonia, we examined the role of BK channels in regulation of skeletal muscle excitability. Intracellular recording revealed slowed repolarization of APs in the absence of BK channels, but this had little effect on pathologic depolarization following induction of myotonia by pharmacologic block of ClC-1 Cl^−^ channels. Force recordings revealed no significant reduction in pharmacologically-induced myotonia in the absence of BK channels. In contrast, the current standard of care (mexiletine), prevented myotonic discharges and greatly improved muscle relaxation times in wild type muscle with pharmacologically-induced myotonia. We conclude that while BK channels contribute to regulation of muscle excitability, block of BK channels, unfortunately, has little potential as therapy for myotonia congenita.

## Materials and methods

### Mice

BK^–/–^ mice were obtained from Andrea Meredith at the University of Maryland and a breeding colony established at Wright State. Wildtype littermates were used as controls. Mice were sacrificed either by guillotine or by using CO_2_ inhalation followed by cervical dislocation. The method of euthanasia had no discernable effect on excitability.

### Force recordings

One EDL muscle per mouse was dissected out intact. The proximal end of the EDL was attached to the lever of the force transducer (300D-305C dual-mode muscle lever, Aurora Scientific, Ontario, Canada) using 5.0 or 6.0 silk suture and a modified Miller’s knot. Optimal length was obtained by measuring the maximum twitch force while lengthening the muscle using a micromanipulator. The recording chamber was continuously perfused with Ringer Solution containing (in mM): NaCl, 118; KCl, 3.5; CaCl_2_, 1.5; MgSO_4_, 0.7; NaHCO_3_, 26.2; NaH_2_PO_4_, 1.7; glucose, 5.5 (pH 7.3–7.4 at 20–22 °C) and equilibrated with 95% O_2_ and 5% CO_2_. Muscles were directly stimulated via platinum electrodes placed on either side of the muscle. A Dagan S-900 Stimulator and S-910 Stimulus Isolation Unit were used for stimulation. Muscle force was recorded and digitized using pClamp10 software (Molecular Devices, San Jose, CA). No filtering was applied to the signal. To induce myotonia, Cl^−^ channels were blocked with 100 µM 9-anthracenecarboxylic acid (9AC) [30].

### Intracellular recording

One EDL muscle per mouse was studied. The same solution used for recording of force was perfused at 20–22 °C. Contraction was prevented by loading muscles with 50 μM BTS (N-benzyl-p-toluenesulfonamide, Tokyo Chemical Industry, Tokyo, Japan, catalogue #B3082) dissolved in DMSO for 45 min prior to recording [22]. Muscle fibers were impaled with 2 sharp microelectrodes filled with 3 M KCl solution containing 1 mM sulforhodamine 101 (Sigma-Aldrich, Catalogue #S7635) to allow for visualization. Electrode resistances were between 10 and 30 MΩ, and capacitance compensation was optimized prior to recording. Action potentials were evoked by a 0.2 ms injection of current ranging from 100 to 1000 nA. Membrane time constant was measured during a 200 ms injection of hyperpolarizing current ranging from 5 to 40 nA. Fibers with resting potentials more depolarized than –74 mV were discarded. Sampling frequency was 50 kHz with a 5 kHz low pass filter.

### Statistics

Nested analysis of variance was used for comparisons of intracellular recordings of muscle fiber properties (R software). Two sample t-tests were performed for mean muscle twitch force measurements with a Welch correction since wild-type twitch force data was not drawn from a normally distributed population. More than two mean force values were compared with a one-way ANOVA and a Bonferroni test using OriginPro 2024 software (OriginLab). Averaged results are expressed as mean ± SD unless otherwise indicated. *p* value < 0.05 and < 0.01 are denoted by one and two stars respectively.

## Results

A previous study suggested that block of BK channels lessens myotonia triggered by block of ClC-1 Cl^−^ channels with 9-anthracenecarboxylic acid (9AC) [19]. It was hypothesized that lessening of myotonia in muscle lacking BK channels (BK^−/−^ muscle) was due to reduction of K^+^ build-up in t-tubules and the resultant decrease in depolarization during repetitive firing [19]. However, intracellular recording was not performed to confirm an effect of BK channels on membrane potential. We are unaware of any studies examining the effect of BK channels on muscle membrane potential.

**Fig. 1:**
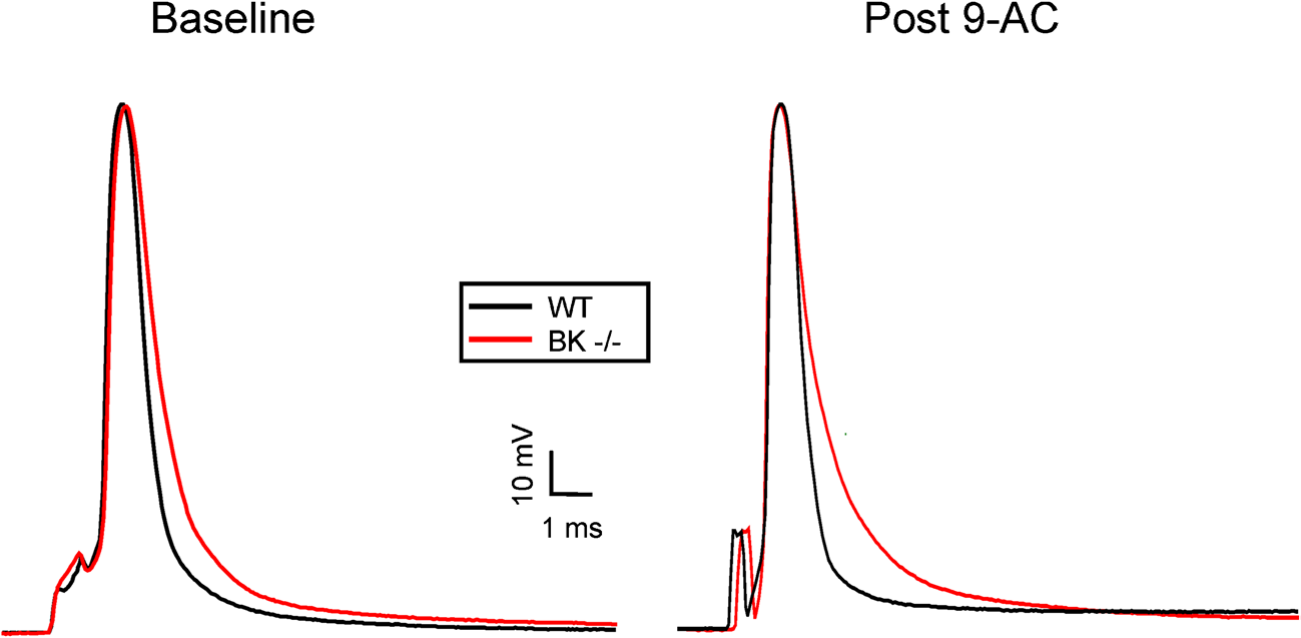
Widening of action potentials in mouse EDL fibers lacking BK channels. Shown superimposed are representative action potentials from WT and BK^−/−^ fibers at baseline and following block of Cl^−^ channels with 9AC. Action potential from WT fibers are shown in black and repolarize more rapidly

Passive properties (resting potential, input resistance, and membrane time constant) as well as the action potential (AP) peak, maximum rate-of-rise (dv/dt up), maximum repolarization rate (dv/dt down), 40% decay time and 80% decay times of single APs were measured in EDL muscle fibers from wild type and BK^−/−^ mice. The 40% and 80% decay times are the times required for the voltage to repolarize from the AP peak to 40% and 80% back to the resting membrane potential, which we have shown to be a reliable and convenient measure of both phases of repolarization in single APs and trains of APs [27]. There were no differences in resting potential, input resistance and time constant between wild type and BK^−/−^ muscle fibers (Table 1), suggesting that BK channels do not contribute to the resting/passive properties of skeletal muscle. While the rising phase and peaks of APs were unchanged in BK^−/−^ fibers, the decay phase was prolonged, suggesting that BK channels contribute to repolarization (Fig. 1, Table 1). Treatment with 100 µM 9AC to block ClC-1 Cl^−^channels was used to model myotonia congenita. There were no differences in resting potential, time constant and input resistance between WT + 9AC and BK^−/−^ + 9AC EDL fibers (Table 1). The slowing of AP repolarization was similar in WT + 9AC and BK^−/−^ + 9AC muscle (Table 1). These data are consistent with BK channels contributing to K^+^ efflux during repolarization and thus K^+^ build-up in t-tubules.

**Table 1:** Slowed repolarization of action potentials in EDL muscle fibers from mice lacking BK channels

	WT (*n* = 6 muscles, 66 fibers)	BK^−/−^ (*n* = 6 muscles, 72 fibers)	WT + 9AC (*n* = 9 muscles, 64 fibers)	BK^−/−^ + 9AC (*n* = 6 muscles, 43 fibers)
Vm (mV)	-82.6 ± 1.7	-83.5 ± 0.7	-82.9 ± 2.8	-83.5 ± 2.7
Time constant (ms)	3.0 ± 0.4	3.3 ± 1.1	11.2 ± 1.1	11.0 ± 0.9
Input resistance (MΩ)	0.29 ± 0.04	0.29 ± 0.05	0.71 ± 0.20	0.91 ± 0.18
AP Peak (mV)	39.7 ± 2.2	39.1 ± 6.2	39.1 ± 3.9	39.8 ± 4.3
dv/dt up (mV/ms)	323 ± 5	316 ± 38	344 ± 35	357 ± 64
dv/dt down (mV/ms)	142 ± 13	111 ± 21**	152 ± 25	113 ± 14**
40% decay (ms)	0.49 ± 0.02	0.60 ± 0.07**	0.47 ± 0.07	0.60 ± 0.08**
80% decay (ms)	1.09 ± 0.06	1.55 ± 0.26**	1.11 ± 0.18	1.81 ± 0.34**

*Vm*  resting membrane potential, *AP* action potential, *dv/dt up*  the maximal rate of depolarization during the rising phase of the action potential, *dv/dt down* the maximal rate of repolarization during the falling phase of the action potential. 40 and 80% decays times = the time for the action potential to repolarize by 40 and 80%. *WT* wild type, BK^−/−^ muscle lacking functional BK channels. * = *p* < 0.05 versus WT in the same treatment group, ** = *p* < 0.01 versus WT in the same treatment group (ANOVA). n represents the number of muscles studied. At least 4 fibers were studied in each muscle. Values are muscle averages ± the standard deviation of muscle averages

**Fig. 2:**
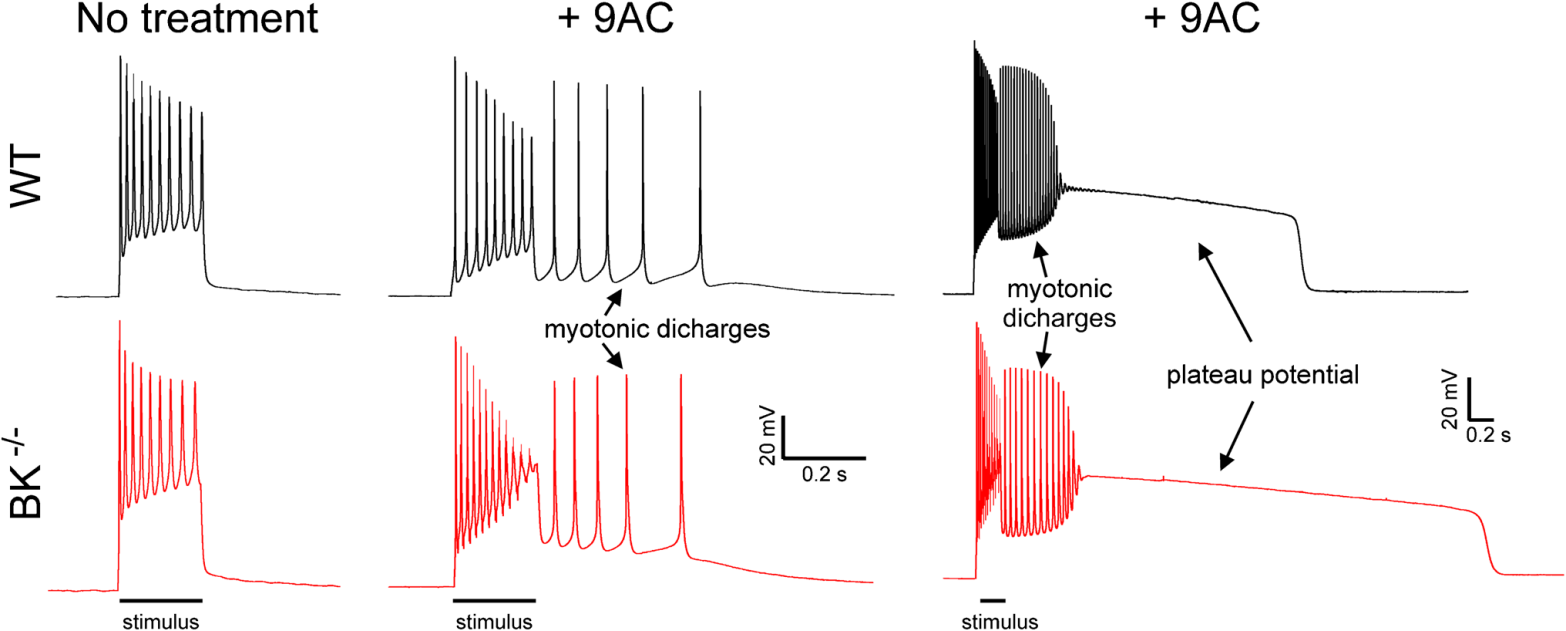
Representative traces from wild type and BK^−/−^ muscle before and following treatment with 9AC to induce myotonia. The leftmost traces show that prior to treatment with 9AC both wild type (WT) and BK^−/−^ fibers fire throughout a 200 ms stimulus, but immediately cease firing when the stimulus ends. The middle traces show that following treatment with 9AC, there are myotonic discharges that can terminate with repolarization. On the right are representative traces in which myotonic discharges terminated with depolarization into a plateau potential

To study the effect of BK channels on excitability of myotonic muscle, intracellular recordings were performed in 9AC-treated fibers stimulated by a 200 ms injection of current. Slowed relaxation of muscle in myotonia congenita is due to both myotonic discharges (involuntary firing of APs) and development of plateau potentials (depolarizations to near -30 mV lasting seconds to minutes) [28, 44](Fig. 2). Severity of the electrophysiological dysfunction was quantified by measuring the percentage of fibers with myotonic discharges and/or plateau potentials, as well as the duration of pathologic depolarization (duration of myotonic discharges + duration of plateau potentials). There was no difference in the percentage of hyperexcitable fibers or the duration of pathologic depolarization between WT + 9AC and BK^−/−^ + 9AC muscle (Table 2). These data suggest BK channels have little effect on pathologic depolarization of myotonic muscle.

**Table 2:** Hyperexcitability in 9AC treated wild type and BK^−/−^ muscle

	WT (*n* = 6 muscles)	BK^−/−^ (*n* = 6 muscle)	WT + 9AC (*n* = 9 muscles)	BK^−/−^ + 9AC (*n* = 6 muscle)
Hyperexcitable fibers	0/66	0/72	64/64	43/43
Duration of pathologic depolarization (s)	NA	NA	2.36 ± 1.13	2.27 ± 1.56
Fibers with plateau potentials	0/66	0/72	58/64	39/43

The duration of pathologic depolarization was defined as the duration of myotonic discharges + the duration of plateau potentials. No differences were statistically significant. Mean values shown are muscle averages ± the standard deviation of muscle averages

**Fig. 3:**
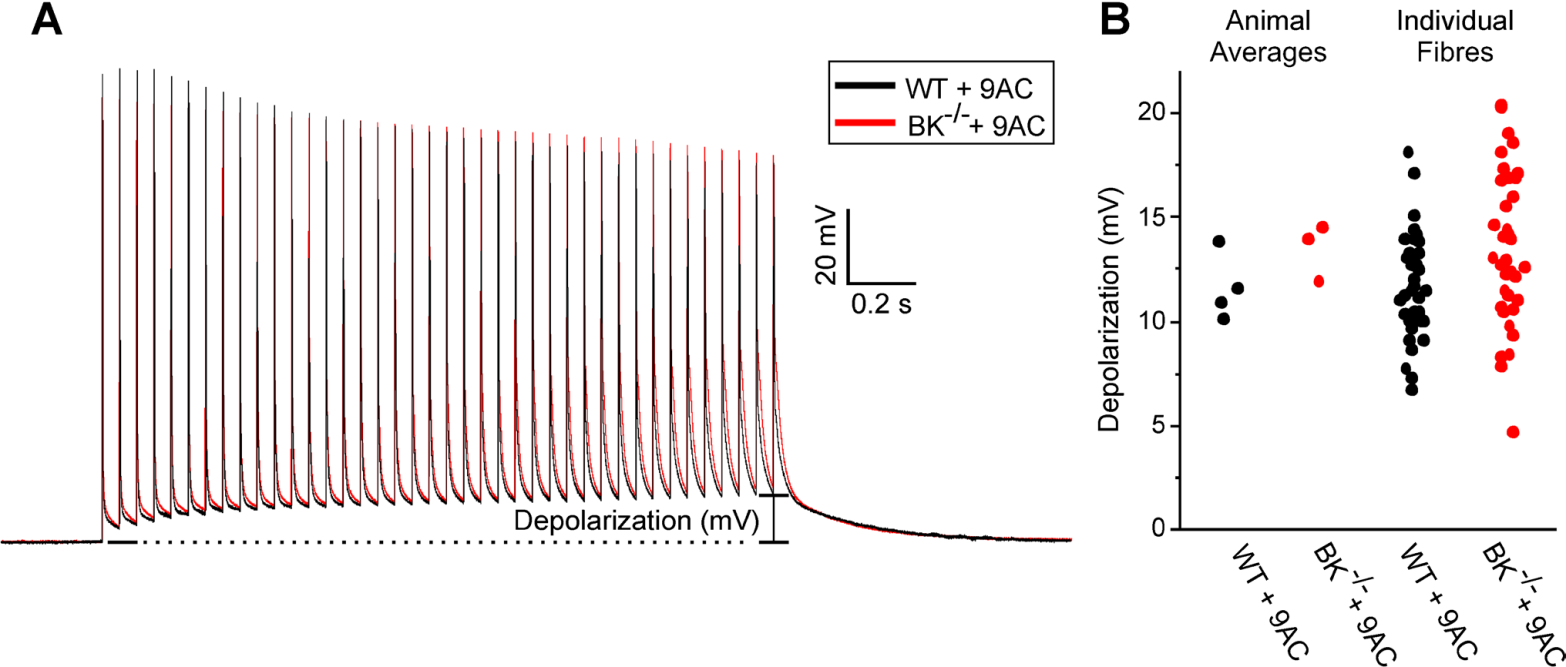
BK channels do not play a role in depolarization of the interspike membrane potential. A) Shown superimposed are representative traces of the response to 2 s of 20 Hz stimulations in WT + 9AC and BK^−/−^ + 9AC EDL muscle fibers treated with 80 nM µ-CTX GIIIA. B) Scatter plots of animal averages and individual fibre depolarization of the interspike membrane potential prior to the 40th action potential in WT + 9AC and BK^−/−^ + 9AC. For WT, *n* = 4 muscles and 37 fibers. For BK^−/−^, *n* = 3 muscles and 35 fibers. Differences were not statistically significant

While there was no effect on the duration of pathologic depolarization, it remained possible that BK channels contribute to K^+^ build-up in t-tubules, which manifests as depolarization of the interspike membrane potential during repetitive stimulation [1, 41]. 9AC-treated fibers were stimulated at 20 Hz for two seconds and depolarization of the interspike interval was measured by subtracting the maximum repolarization before the 40th action potential from the membrane potential prior to the first action potential. To eliminate myotonic discharges, which add the confound of additional action potentials during stimulation, 80 nM of the muscle specific Na^+^ channel blocker µ-CTX GIIIA was added [12]. We have found this concentration of µ-CTX GIIIA eliminates myotonia without having a significant impact on the build-up of depolarization of the interspike membrane potential (supplemental Fig. 1). The hypothesis was that muscle lacking BK channels would have less depolarization of the interspike membrane potential. Surprisingly, there was a trend towards increased depolarization of the interspike membrane potential in BK^−/−^ + 9AC vs WT + 9AC muscles, which would worsen myotonia in BK^−/−^ muscle, but the difference was not significant (Fig. 3). These data suggest that despite playing a role in AP repolarization, BK channels do not contribute to accumulation of K^+^ in t-tubules.

Given the lack of effect of BK channels on pathologic depolarization, we performed force recordings to determine whether we could replicate the results of Hoppe et al. in which myotonia was less in BK^−/−^ muscle [19]. We measured twitch force, similar to Hoppe et. al., as well as tetanic force at 20 and 60 Hz. After setting optimal length, 9 WT and 5 BK^−/−^ EDL muscles were stimulated with the following protocol: 0.1 Hz for 20 pulses to record twitches, 5 min rest, 20 Hz for 40 pulses, 5 min rest, and 60 Hz for 100 pulses. Then, the muscles rested (no stimulation) in the presence of 9AC or 9AC + mexiletine for 40 min: 6 WT and 5 BK^−/−^ EDL muscles were exposed to 100 µM 9AC and 3 WT EDL muscles were exposed to 100 µM 9AC + 20 µM mexiletine. After exposure to the drug(s), force was measured during the same 0.1, 20, and 60 Hz stimulation protocol.

**Fig. 4:**
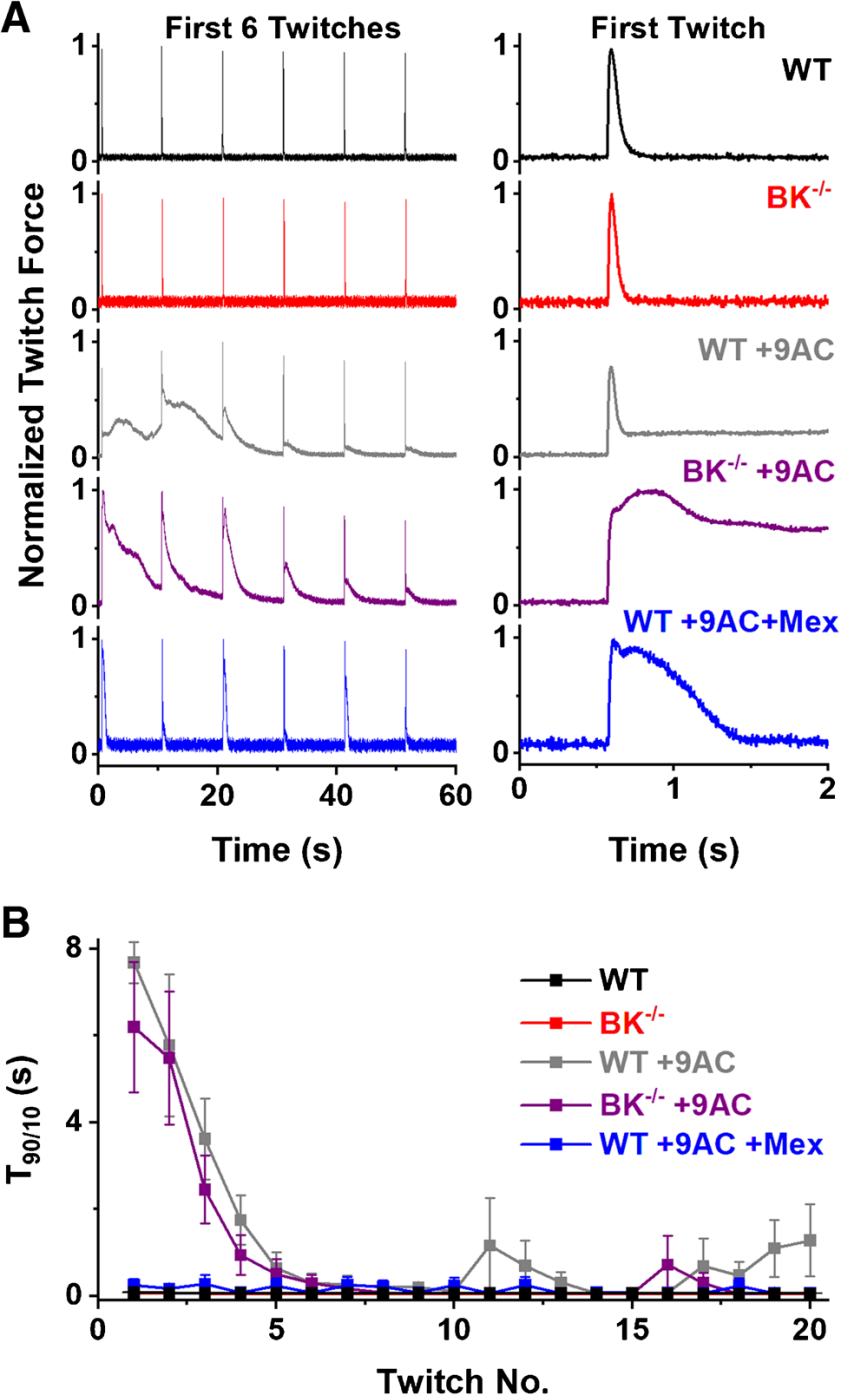
BK channels have little effect on myotonia at low rates of repetitive firing. A) On the left are representative traces of the force generated in response to the first 6 stimulation delivered at 0.1 Hz from the 5 different treatment groups. Shown on the right is the first twitch from each trace on the left, on a higher resolution time scale. B) Plot of the 90% to 10% of the maximal value (T_90/10_) time during the 0.1 Hz train. Shown are the mean ± SD for each of the 20 stimuli for each treatment group. Mex = mexiletine. The red plot of myotonia in BK^−/−^ muscle without 9AC is hidden behind the plot for WT without 9AC

Representative traces of the first 6 twitches from WT, BK^−/−^, WT + 9AC, BK^−/−^ + 9AC, and WT + 9AC + mexiletine are shown in Fig. 4A. As done in Hoppe et. al., twitches were analyzed for the time required for force to decrease from 90 to 10% of max (T_90/10_). In the absence of 9AC, the T_90/10_ relaxation time of the first twitch in the 0.1 Hz train of WT muscle (72.3 ± 12.9 ms, *n* = 9) and BK^−/−^ muscle (60.2 ± 13.3, *n* = 5) were not significantly different; although there was a tendency toward a shorter twitch time in muscle lacking BK channels (*p* = 0.077, t-test). This differed from the results of Hoppe et al., in which the T_90/10_ relaxation time was near 100 ms for wild type and 150 ms for EDL muscle lacking BK channels [19]. Following block of ClC-1 channels with 9AC, there was a marked prolongation of the T_90/10_ relaxation time in both groups, with no significant difference between WT (7686.3 ± 1158.8 ms, *n* = 9) and BK^−/−^ (6196.7 ± 3374.3) (*p* = 0.391). High variability in the BK^−/−^ data prevented the apparently lower BK^−/−^ T_90/10_ relaxation time from being significantly less than that of WT. While similar in direction, the magnitude was less than that seen by Hoppe et al. In contrast, treatment of myotonic muscle with mexiletine (WT + 100 µM 9AC + 20 µM mexiletine), the current standard of care, dramatically reduced myotonia. The T_90/10_ relaxation time of WT muscle plus 100 µM 9AC that was treated with 20 µM mexiletine (257.0 ± 249.6 ms, *n* = 3) was significantly less than that of WT + 9AC (*p* = 0.002) and BK-/- + 9AC (*p* = 0.002) muscle (ANOVA with Bonferroni comparison).

We also examined the T_90/10_ values for all 20 stimuli in the 0.1 Hz train (Fig. 4B). After 6 twitches, the T_90/10_ values for WT and BK^−/−^ EDL muscles treated with 9AC had decreased to near control (no 9AC) levels, which is likely attributable to the “warm-up” phenomenon [29]. Consistent with Hoppe et. al., the T_90/10_ values for BK^−/−^ appeared slightly less than WT during this activity-induced decline in myotonia. However, no elevation in T_90/10_ values was evident in the WT + 9AC + mexiletine group, highlighting the efficacy of mexiletine in treating myotonia (Fig. 4B).

**Fig. 5:**
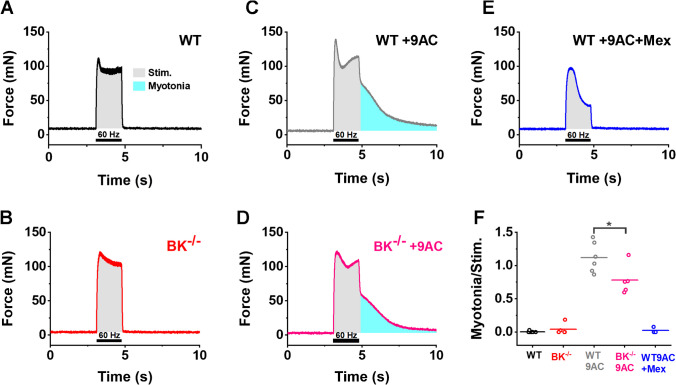
Knockout of BK channels has little effect on myotonia at high rates of repetitive firing. **A-E** Representative traces of the force generated in response of EDL muscles to 1.7 s of 60 Hz stimulation, black horizontal bars indicate the stimulation time. The integral of force during stimulation is shaded light gray and the integral for myotonic force after stimulation is shaded cyan. **A, B** In the absence of 9AC treatment, both wild type and BK^−/−^ muscle rapidly relaxed following termination of stimulation. **C, D** Following 9AC treatment, both wild type and BK^−/−^ muscle had several seconds of myotonic contraction following termination of stimulation. **E** Treatment with 20 µM Mexiletine caused rapid relaxation of 9AC treated muscle. **F** Scatter plot of the integral of myotonic force normalized to the integral of force during stimulation for each muscle studied in all five treatment groups. The horizontal lines represent the mean. * indicates *p* < 0.05

Human motor units in vivo fire at rates greater than 8 Hz [26] and K build-up in t-tubules would be expected to worsen with faster rates of repetitive firing. Thus, we also stimulated the EDL at 20 and 60 Hz. As observed for twitch force, mexiletine was substantially better at reducing myotonia compared to BK^−/−^ muscle. For simplicity, we show the data for only 60 Hz (Fig. 5). We detected little to no myotonic force following stimulation in WT and BK^−/−^ muscle (Fig. 5A, B). In contrast, both WT + 9AC and BK^−/−^ + 9AC muscle exhibited clear myotonic force following stimulation (Fig. 5C, D). Strikingly, exposure of WT + 9AC to 20 µM mexiletine (WT + 9AC + Mex) almost completely eliminated myotonic contraction following stimulation (Fig. 5E).

To quantify severity of myotonia, we measured the integral of force with respect to time, as this measure incorporates both force and duration of contraction. The integral of force following stimulation (myotonia, cyan shading) was divided by the integral of force during the 60 Hz stimulation (gray shading) to normalize for differences in strength (Fig. 5F). Myotonia was significantly more severe in WT + 9AC and BK^−/−^ + 9AC compared to WT, BK^−/−^ and WT + 9AC + Mex (*p* < 0.0001, one-way ANOVA, Bonferroni). There was also significantly less myotonia in BK^−/−^ + 9AC compared to WT + 9AC (*p* = 0.008, one-way ANOVA, Bonferroni), which is consistent with the findings of Hoppe et al. that inhibition of BK channels may reduce myotonia. However, similar to the results of stimulation at 0.1 Hz, treatment with 20 µM mexiletine caused a much greater reduction in myotonia than knockout of BK channels (Fig. 5), as there was no significant difference in the degree of myotonia in WT, BK^−/−^ and WT + 9AC + Mex (one-way ANOVA, Bonferroni).These data suggest the current standard of care is superior to elimination of current through BK channels as therapy for myotonia.

## Discussion

We demonstrated that BK channels contribute to repolarization of mouse EDL muscle action potentials (APs). Despite a role in regulation of muscle excitability, knockout of BK channels had little effect on myotonia triggered by block of muscle Cl^−^ channels with 9AC. Our data argue against a major role of BK channels in triggering of myotonia by worsening build-up of K^+^ in t-tubules. We conclude that blocking BK channels is unlikely to provide effective therapy for myotonia.

### BK channels contribute to repolarization of action potentials in skeletal muscle.

BK channels are known to play a central role in the regulation of excitability in a number of cell types [6, 33, 45]. Two of the earliest studies of BK channel function were performed in skeletal muscle and demonstrated the presence of functional channels in the sarcolemma [4, 32]. BK^–/–^ mice exhibit significant motor dysfunction, including problems with gait and weak grip [9, 18, 24, 34, 40, 42]. Despite the wealth of knowledge regarding the effect of BK channels on excitability and the knowledge that BK channels are present in the sarcolemma, there have been few studies of the role of BK channels in regulation of muscle excitability. A study in frog muscle found that activation of BK channels pharmacologically caused slight hyperpolarization of the resting membrane potential and decreased AP amplitude [11]. We are unaware of studies in mammals to determine the function of BK channels in muscle.

We did not find any difference in resting membrane potential or membrane resistance between wild type and BK^−/−^ muscle. These findings fit well with the understanding that BK channels are voltage- and Ca^2+^-activated, such that they would not be expected to be open at rest [33, 46]. There was slowing of AP repolarization in BK^−/−^ muscle, suggesting BK channels are activated during APs and contribute to repolarization. It has been demonstrated in neurons that the effects of BK channels on AP repolarization plays a critical role in regulation of repetitive firing [33]. Based on the effect of BK channel knockout on properties of single APs in muscle, it seemed reasonable to explore their role in regulation of myotonia.

### The role of BK channels in triggering myotonia.

An important contributor to generation of myotonic discharges is depolarization of the interspike interval, which is caused by a combination of accumulation of K^+^ in t-tubules and activation of a small, non-inactivating Na^+^ current [25]. Normally, ClC-1-mediated Cl^−^ current offsets the depolarizing influence of K^+^ accumulation [1, 3, 31, 35, 36]. During repeated firing of APs, build-up of K^+^ in t-tubules shifts the K^+^ Nernst potential to more depolarized values and can trigger myotonic discharges [1, 2, 8, 41]. Given the importance of K^+^ build-up in triggering myotonic discharges, manipulation of K^+^ channels is an appealing approach to treating myotonia congenita. It would complement the current approach of blocking Na^+^ channels [7, 23]. However, in order to develop novel therapy via manipulation of K^+^ channels, it is necessary to understand the role of various K^+^ channels in K^+^ build-up in t-tubules.

The largest K^+^ current in skeletal muscle is carried by Kv (delayed rectifier) channels, which serve to repolarize the membrane potential following firing of an AP [5, 13]. There is a marked reduction in Kv current in skeletal muscle following osmotic decoupling of the t-tubules from the surface membrane, consistent with the possibility that the majority of Kv channel are located in the t-tubules [13]. It thus seems likely that K^+^ build-up in the mouse EDL muscle is mediated primarily by K^+^ flow through Kv channels. However, our finding that repolarization of APs is slowed in muscle lacking BK channels suggests there is also significant K^+^ efflux via BK channels. This is consistent with the finding that BK channels are expressed at significant levels in skeletal muscle [14, 38, 39]. Hoppe et al. demonstrated via immunohistochemistry that BK channels are present in t-tubules [19]. Taken together, these data suggest that BK channels are in the t-tubules where they might contribute to the K^+^ build-up.

We found no change in the interspike membrane potential between 9AC treated wild type and BK^−/−^ EDL muscles during 20 Hz stimulation. One possibility is that there is compensatory upregulation of Kv channels in BK^−/−^ EDL muscle. However, compensation seems unlikely to explain the lack of effect on myotonia. Hoppe et al. found the same effect on myotonia with acute block of BK channels and knockout of BK channels [19]. Furthermore, our data showing slowed repolarization of APs suggests reduced K^+^ conductance slows repolarization of APs in BK^−/−^ EDL muscle. Thus, if there is compensation, it is partial.

We favor the possibility that reducing K^+^ current during the falling phase of the AP did not reduce K^+^ build-up. Repolarization of the membrane potential during the falling phase of APs is accomplished by reversing charge build-up on the muscle membrane capacitor that occurred during the rising phase of the AP. To repolarize from + 30 mV to -85 mV requires the same amount of K^+^ exit into t-tubules regardless of K^+^ conductance. Reduction of K^+^ conductance prolongs the time it takes to repolarize, but does not change the net K^+^ flux into t-tubules required for repolarization. If K^+^ accumulation in t-tubules is not altered by changes in K^+^ channel density in t-tubules, this suggests that attempts to lessen K^+^ build-up by blocking K^+^ channels will be ineffective. This does not mean that targeting K + build-up in t-tubules cannot be used as an approach to therapy for myotonia. It may be possible to treat myotonia via stimulation of the Na^+^, K^+^ ATPase, which plays a crucial role in reuptake of K^+^ from t-tubules following firing of APs [10]. Stimulation of the Na^+^, K^+^ ATPase has been suggested to lessen severity of attacks of weakness in hyperkalemic periodic paralysis [43].

### Activation, rather than block, of K + channels as therapy for myotonia

The Kv channel agonist retigabine lessens myotonia [15, 37]. The mechanism underlying efficacy of retigabine is likely that it opposed depolarization towards AP threshold, which is driven by a small, non-inactivating persistent Na^+^ current [15, 25]. K^+^ channels on the sarcolemma do not experience the same degree of depolarized shift in the Nernst potential of K^+^ during repetitive firing of APs as K^+^ channels in t-tubules. Thus, if some Kv channels are present on the sarcolemma, activation of those channels would oppose depolarization. One concern with use of retigabine or other K^+^ channel openers had been that they might worsen K^+^ build-up. Our current work suggests this is not an issue as activation of K^+^ channels in t-tubules is unlikely to worsen K^+^ build-up. We conclude that activation, rather than block, of K + channels is the more promising approach to development of novel therapy for myotonia.

We came to a different conclusion regarding the efficacy of block/knockout of BK channels in treating myotonia than Hoppe et al. {Hoppe, 2020 #8381}. They concluded block of BK channels has potential as therapy for myotonia congenita, whereas we conclude activation rather than block of K^+^ channels is more likely to be effective. The difference is not due to a discrepancy in data. Both studies found the time to relaxation of muscle following treatment with 9AC to block Cl^−^ channels was shorter in muscle lacking BK channels. The difference in our study was smaller, but was still statistically significant. The biggest difference is that we performed intracellular recording and found no decrease in the tendency toward depolarization during repetitive stimulation of BK^−/−^ muscle treated with 9AC to induce myotonia. Our data suggest faster relaxation of 9AC treated BK^−/−^ muscle is not due to lessening of hyperexcitability. Rather it is due to some other effect of BK channels. We previously found that knockout of BK channels alters the function of the neuromuscular junction via a likely reduction in presynaptic Ca^2+^ current that is independent of K^+^ current flow through the channel [42]. It is possible that knockout of BK channels alters Ca^2+^ handling in muscle in a way that is independent of effects on membrane potential. We hypothesize knockout of BK channels speeds relaxation of muscle without affecting myotonic discharges.

## Supplementary Information

Below is the link to the electronic supplementary material.

Supplementary file1 (DOCX 131 KB)

## Data Availability

Data is provided within the manuscript.

## Abbreviations

AP: Action potential
EDL: Extensor digitorum longus
Vm: Resting membrane potential
WT: Wild type
9AC: 9-Anthracenecarboxylic acid
