# Supplementary material for: BK channels promote action potential repolarization in skeletal muscle but contribute little to myotonia
Source: Pflugers Arch. 2024 Aug 16;476(11):1693–702. doi: 10.1007/s00424-024-03005-z (PMC11461784; doi:10.1007/s00424-024-03005-z)
Supplement: Supplementary file 1 — Supplementary file1 (DOCX 131 KB) [file 424_2024_3005_MOESM1_ESM.docx]

Supplemental Fig 1- 80 nM µ-CTX GIIIA does not affect depolarization of the interspike membrane potential of 9AC treated diaphragm muscle fibres. Shown is a box and whisker plot of the depolarization of the interspike membrane potential at the end of 40 action potentials triggered at 20 Hz with and without uCTX. N= 17 fibers from 3 muscles for both data sets.
